# Cluster Analysis of Clinical Data Identifies Fibromyalgia Subgroups

**DOI:** 10.1371/journal.pone.0074873

**Published:** 2013-09-30

**Authors:** Elisa Docampo, Antonio Collado, Geòrgia Escaramís, Jordi Carbonell, Javier Rivera, Javier Vidal, José Alegre, Raquel Rabionet, Xavier Estivill

**Affiliations:** 1 Genomics and Disease Group, Centre for Genomic Regulation (CRG), Barcelona, Spain; 2 Universitat Pompeu Fabra (UPF), Barcelona, Spain; 3 Centro de Investigación Biomédica en Red en Epidemiología y Salud Pública (CIBERESP), Barcelona, Spain; 4 Fibromyalgia Unit, Rheumatology Service, Hospital Clínic, Barcelona, Spain; 5 Fibromyalgia Unit, Rheumatology Service, Parc de Salut Mar; and Hospital del Mar Research Institute, Barcelona, Spain; 6 Rheumatology Unit, Instituto Provincial de Rehabilitación, Hospital Universitario Gregorio Marañón, Madrid, Spain; 7 Rheumatology Unit, Hospital General de Guadalajara, Guadalajara, Spain; 8 Chronic Fatigue Syndrome Unit, Hospital Vall d’Hebron, Barcelona, Spain; 9 Fibromyalgia and Chronic Fatigue Syndrome, Spanish Genetic and Clinical Data Bank Group, Foundation Fibromyalgia and Fatigue, Barcelona, Spain; Iran University of Medical Sciences, Iran (Republic of Islamic)

## Abstract

**Introduction:**

Fibromyalgia (FM) is mainly characterized by widespread pain and multiple accompanying symptoms, which hinder FM assessment and management. In order to reduce FM heterogeneity we classified clinical data into simplified dimensions that were used to define FM subgroups.

**Material and Methods:**

48 variables were evaluated in 1,446 Spanish FM cases fulfilling 1990 ACR FM criteria. A partitioning analysis was performed to find groups of variables similar to each other. Similarities between variables were identified and the variables were grouped into dimensions. This was performed in a subset of 559 patients, and cross-validated in the remaining 887 patients. For each sample and dimension, a composite index was obtained based on the weights of the variables included in the dimension. Finally, a clustering procedure was applied to the indexes, resulting in FM subgroups.

**Results:**

Variables clustered into three independent dimensions: “symptomatology”, “comorbidities” and “clinical scales”. Only the two first dimensions were considered for the construction of FM subgroups. Resulting scores classified FM samples into three subgroups: low symptomatology and comorbidities (Cluster 1), high symptomatology and comorbidities (Cluster 2), and high symptomatology but low comorbidities (Cluster 3), showing differences in measures of disease severity.

**Conclusions:**

We have identified three subgroups of FM samples in a large cohort of FM by clustering clinical data. Our analysis stresses the importance of family and personal history of FM comorbidities. Also, the resulting patient clusters could indicate different forms of the disease, relevant to future research, and might have an impact on clinical assessment.

## Introduction

Fibromyalgia (FM) is a disorder characterized by widespread pain and accompanying symptoms such as fatigue, disturbed sleep and variable degrees of anxiety and depression [1]. It is a very heterogeneous disease, including different types of symptoms of different systems. In fact, it has even been defined as an overlap of syndromes and symptoms rather than as a discrete entity [2].

Diagnosis of fibromyalgia is based on clinical features. Despite the development of new clinical diagnostic criteria [3], [4], it is difficult to define and assess the severity of FM, due to its multidimensional nature and the lack of specific disease markers [5]. In addition, the heterogeneity of FM hinders the characterization of the interactions among its clinical features, and their relationship with treatment outcome. Thus, many investigators in the field have suggested that there is a need for empirically derived groups that could help to tailor more specific therapies to each patient [6] and quantify health care usage [7].

One of the most characteristic methods for grouping symptoms of a given disorder is cluster analysis. It consists of a multivariate statistical technique that evaluates the degree of similarity among heterogeneous variables in order to identify related groups of variables based on these similarities [8]. Cluster analysis has been widely used to identify patient-relevant clinical features to be used in patient orientated management strategies, especially in the oncology field. It has also been applied to other disorders in an effort to understand the relationships among clinical features and outcome variables. In fact, some studies have already attempted to define FM subgroups by performing cluster analysis of FM symptoms. These studies differed on sample sizes, variables studied and methods used. In most cases, cluster analysis was used to categorize FM patients based on somatic or psychological symptoms [9], [10], quantitative sensory testing [11] or pressure-pain thresholds and psychological factors [12]. A recent study tried to discern clinically relevant subgroups across psychological and biomedical domains [13], while another attempted to identify clusters of clinical features meaningful to FM patients that corresponded to their treatment priority goals in the context of desired improvement [2]. Finally, a recent work [14] indicated the existence of two latent dimensions underlying FM symptomatology: FM core symptoms and distress. Most of these studies have been performed in small cohorts taking into account few clinical variables, and using clustering to group either variables or patients. Only three studies were performed in large cohorts [7], [10], [15]. In these studies, patients were assessed through web based methods with the purpose to evaluate patients perception of symptoms management [15] or to examine differences among FM subgroups in healthcare utilization [7]. Despite the various attempts at classification, there is yet no clear subgrouping of patients or clinical variables that will help in the management of the disease.

In the present study, we want to analyze the co-occurrence in the patients of the clinical variables collected in a large cohort of FM cases, and identify groups of clinical features that would enable to distinguish subgroups of FM patients. The main objective is to describe possible subgroups of patients that may be found in the clinical practice, which may help to understand the disease. We will do this by building a set of dimensions of clinical data, with the inclusion of personal and family comorbidities in addition to physical and psychological symptoms, and then testing its validity in the identification of FM subtypes. We have used a large cohort of 1,446 FM cases, very well characterized at the clinical level, through physician direct interview and physical examination in specialized fibromyalgia units.

## Materials and Methods

### Samples

Data for 1,446 FM patients was obtained from the Fibromyalgia and Chronic Fatigue Syndrome Spanish Genetic and Clinical Data Bank (FFSGCDB) [16] (www.bancoadn.org). The biological and clinical data were collected at the FM units of five Spanish Hospitals. Patients fulfilling the 1990 ACR criteria [1] were selected for inclusion in the study and subsequently evaluated by a set of physicians trained in the evaluation of FM patients. An initial set of 559 unrelated FM cases was considered for the analysis; the same cluster analysis was performed in a second set of 887 cases. The analysis of the relationship between clustered groups and severity of clinical core variables was performed in the entire cohort of 1,446 patients.

### Ethics

All patients were of Spanish Caucasian origin and had signed informed consent before enrollment. The ethics committee at all recruitment centers, Hospital del Mar (Barcelona) Hospital Clínic i Provincial (Barcelona), Hospital de la Vall d’Hebrón (Barcelona), Hospital Gregorio Marañón (Madrid) and Hospital General de Guadalajara (Guadalajara), approved the project.

### Variables

Data collection followed a standard protocol of questionnaires and physical examination that were recorded by principal investigators of FFSGCDB. It included collection of demographic variables (age, marital status, educational level, and occupational status), family and personal history of diseases, FM clinical features, fulfillment of CFS criteria, and treatments. Core measures of FM symptoms and comorbidities were assessed by different Spanish validated scales: pain and fatigue visual analogue scale (VAS), number of tender points [17], Hospital Anxiety and Depression Scale (HAD) [18], [19], Pittsburg Sleep Quality Index (PSQI) [20], Fibromyalgia Impact Questionnaire (FIQ) [21], Fatigue Impact severity Scale (FIS) [22], [23] and Quality of Life survey (SF-36) [24], [25].

After exclusion of treatment and socio-demographic variables, 48 variables were selected for the clustering analysis, based on clinical experience, including symptoms defined in the literature and somatic symptoms used in the new diagnostic criteria (List S1) [3].

These variables can easily be recorded in routine diagnostic procedures: 12 of them are related with personal and family history of medical or psychopathological comorbidities; 4 variables are related to disease evolution; 10 variables correspond to clinical scales and the remaining 22 variables are somatic symptoms.

Cluster analysis with an admixture of continuous and dichotomous variables lead to a clustering of the variables upon their nature. For this reason and taking into account that 75% of the variables considered in the study were dichotomous, the non-dichotomous variables were transformed into binary types (0 = mild; 1 = severe), simplifying the distance analysis but taking into account the severity of the variable. For symptoms (dichotomous variables), the presence of the symptom was codified as 1 and the absence as 0. For continuous variables, the median was considered as a robust cut-off value, as the distribution of the quantitative variables was asymmetrical (Figure S1). For scales, values below the median were codified as 0 and values above the median as 1, while for age of onset, as a younger age of onset (≤38 years) is considered more severe, the codification was reversed. Variables included in the cluster analysis, as well as their medians (interquartile range), are summarized in List S1 and Table 1.

**Table 1:** Summary of the three different dimensions that emerged after cluster analysis.

VARIABLE	VALUES	WEIGHT	NEIGHBOUR
**DIMENSION 1: Symptoms and their characteristics**			
Widespread pain	0 = No 1 = yes	0.417062	3
Muscle weakness	0 = No 1 = yes	0.403494	3
Post exercise fatigue	0 = No 1 = yes	0.401630	3
Morning stiffness	0 = No 1 = yes	0.386802	3
Muscular contractures	0 = No 1 = yes	0.370318	3
Concentration problems	0 = No 1 = yes	0.363702	3
Memory complaints	0 = No 1 = yes	0.343472	3
Onset	0 = Progressive 1 = Sudden	0.327471	3
Sleep Disturbances	0 = No 1 = yes	0.295485	3
Forgetfulness	0 = No 1 = yes	0.232666	3
Migratory joint pain	0 = No 1 = yes	0.232439	3
Headache	0 = No 1 = yes	0.196143	3
Pain subtle movements impairment	0 = No 1 = yes	0.192320	3
Intestinal dysfunction	0 = No 1 = yes	0.188124	3
Visual accommodation impairment	0 = No 1 = yes	0.134756	3
Trigger	0 = No 1 = yes	0.128889	3
Dizziness	0 = No 1 = yes	0.106305	3
Excessive Perspiration	0 = No 1 = yes	0.103443	3
Months of pain (96; p_25_∶48; p_75_∶156)	0≤96 1≥96	0.097555	3
Personal history of chronic pain	0 = No 1 = yes	0.081029	3
Palpitations	0 = No 1 = yes	0.079645	3
Age of onset (38; p_25_∶30; p_75_∶45)	0≥38 1≤38	0.039180	3
**DIMENSION 2: Personal and family comorbidities**			
Posttraumatic stress disorder	0 = No 1 = yes	0.574638	3
Personality disorders	0 = No 1 = yes	0.573018	3
Family history of autoimmune disorders	0 = No 1 = yes	0.555378	3
Family history of chronic fatigue syndrome	0 = No 1 = yes	0.554531	3
Panic attacks	0 = No 1 = yes	0.491861	3
Family history of fibromyalgia	0 = No 1 = yes	0.466244	3
Blackouts	0 = No 1 = yes	0.456721	3
Facial oedema	0 = No 1 = yes	0.432674	3
Connective disorder	0 = FM 1 = FM+CFS	0.395716	3
Adjustment disorder	0 = No 1 = yes	0.363677	3
Previous Personal history psychopathology	0 = No 1 = yes	0.321596	3
Major depression	0 = No 1 = yes	0.295372	3
Family history of chronic pain	0 = No 1 = yes	0.188152	3
Impaired urination	0 = No 1 = yes	0.155414	3
Spine osteoarthritis	0 = No 1 = yes	0.116031	3
Life quality SF-36 physical subscale (27; p_25_∶22; p_75_∶32)	0≥27 1≤27	0.071334	3
Life quality SF36 mental subscale (35; p_25_∶25; p_75_∶48)	0≥35 1≤35	0.004091	1
**DIMENSION 3: Scales**			
HAD depression subscale (10; p_25_∶7; p_75_∶14)	0≤10 1≥10	0.266515	2
Fibromyalgia Impact Questionnaire (FIQ) (74.66; p_25_∶63.05; p75∶84.25)	0≤74.66 1≥74.66	0.249480	1
Fatigue Impact Scale (FIS) (66; p_25_∶56.50; p_75_∶75.00)	0≤66 1≥66	0.241993	1
Pain level (VAS 1–10 cm) (7.5; p_25_∶6.5; p_75_∶8.5)	0≤7.5 1≥7.5	0.196313	2
Pittsburgh Sleep Quality Index (PSQI) (14; p_25_∶11; p_75_∶17)	0≤14 1≥14	0.194950	1
Fatigue level (VAS 1–10 cm) (8; p_25_∶6.4; p_75_∶9)	0≤8 1≥8	0.191182	1
HAD anxiety subscale (12; p_25_∶8; p_75_∶15)	0≤12 1≥12	0.105136	1
Number of Tender Points (13; p_25_∶13; p_75_∶18)	0≤16 1≥16	0.055568	1
Trembling	0 = No 1 = yes	0.043791	1

The variables included in each dimension are listed and sorted by their weighted contribution. For continuous variables, the median was used as cut-off value for binary codification. Weight represents the relationship between the similarity of the variable to the other variables of the dimension and its similarity to the remaining variables. Neighbor represents the closest dimension, excluding the one in which the variable was included.

### Statistical Analysis

#### Building variable’s dimensions

The underlying dimensions of FM were evaluated using *partitioning cluster analysis*. This is a method to partition data into meaningful subgroups when the number of subgroups and other information about their composition may be unknown [26]. The basic procedure behind partitioning cluster analysis is to construct subgroups with homogeneous objects, which in our study are clinical variables, based on a well-defined proximity measure. Given the non-continuous nature of our variables, we used the Gower’s general similarity measure [27]. We used a robust approach called *position around medoids* (PAM), where group membership depends on proximity to an actual observation (medoid) instead of proximity to an average object (centroid). The number of clusters or subgroups was determined using silhouette plots and Calinski’s index. The names for each of the subgroups were determined post-hoc in an attempt to characterize the nature of each FM dimension.

The clustering was constructed in an initial sample of 559 FM patients, and a replication of the analysis was performed in a second sample of 887 patients (see Figure S2).

#### Construction of samples subgroups

The clustering tool to define the underlying FM dimensions was used to further construct patient synthetic indexes based on the clinical features composition of each FM dimension. The values of the synthetic indexes were calculated from linear functions (one per each dimension, see Methods S1) that weight the dichotomous variables that constitute the specific dimension. The weighting factor is based on the silhouette value of the specific variable in the cluster to which it is assigned. The silhouette values take meaningful values from 1 (well assigned) to −1 (closer to variables assigned to neighboring clusters), where 0 indicates no clear assignment. The resulting values of the synthetic indexes result into continuous measures for each FM dimension.

The synthetic indexed values calculated per each sample and dimension where then used to find patient subgroups. Given now the continuous nature of the measures we used the K-means clustering procedure to group patients with similar FM profiles into three meaningful subgroups.

ANOVA analyses were used to compare the behavior of the 11 measured clinical scales in the defined FM subgroups, and are shown in table 2.

**Table 2:** Means ± standard error of the mean (SEM) of different pain, psychiatric and quality of life scores in each of the fibromyalgia clinical subgroups.

VARIABLE	Cluster 1	Cluster 2	Cluster 3	P-value
	FM LowLow (n = 283)	FM HighHigh (n = 357)	FM HighLow (n = 758)	
Fibromyalgia Impact Questionnaire (FIQ)	59.46±1.16	77.69±0.72	73.16±0.52	p<0.001
Fatigue Impact Scale (FIS)	65.10±0.72	69.02±0.57	53.37±0.6	p<0.001
Pain level (VAS 1–10 cm)	6.30±0.12	7.88±0.08	7.43±0.06	p<0.001
Fatigue level (VAS 1–10 cm)	6.33±0.14	7.98±0.09	7.43±0.07	p<0.001
Number of Tender Points	13.54±0.21	15.54±0.14	15.24±0.1	p<0.001
Life quality SF-36 physical subscale	30.7±0.62	26.71±0.36	26.86±0.28	p<0.001
Life quality (SF36) mental	42.12±0.86	31.73±0.67	36.08±0.49	p<0.001
HAD anxiety subscale	9.54±0.27	13.03±0.23	12.12±0.16	p<0.001
HAD depression subscale	7.48±0.27	11.48±0.24	10.39±0.16	p<0.001
Pittsburgh Sleep Quality Index (PSQI)	11.28±0.26	15.35±0.18	14.27±0.14	p<0.001
Years of disease evolution	11.14±0.57	13.60±0.57	11.63±0.67	0.07

P-value of multinomial analysis.

All analyses were performed in the R environment (R software version 2.14.1), using the *cluster* and *fpc* packages.

## Results

1,446 FM samples of Caucasian origin were included in the study. Of these, 97.2% were women with a mean age of 49±10 years; more than two thirds (70.5%) were married; more than a third (37.5%) had a high school degree and an additional 19,3% had gone to university; and 80% of the patients had a paid employment.

**Figure 1:**
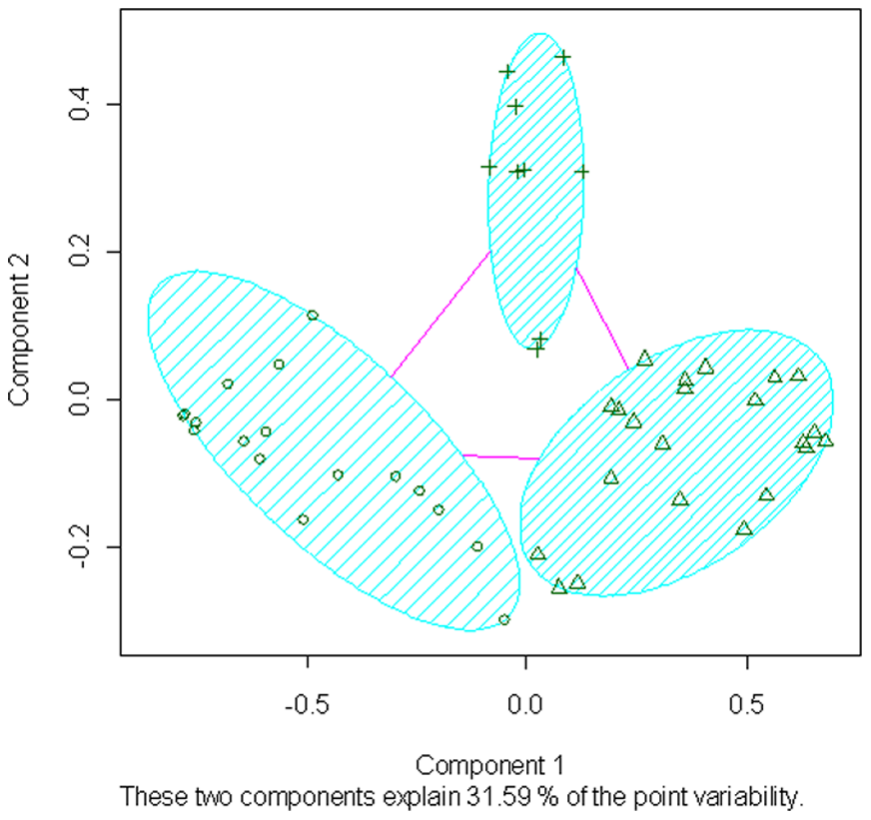
Clustering of variables into three dimensions. Crosses, triangles and circles represent variables assigned to each of the dimensions. X and Y axis represent the two first principal components of a PCA analysis used to reduce the dimensionality of the data in order to be able to illustrate the clustering results.

In the first dataset of 559 unrelated FM cases, the selected 48 clinical variables clustered into three independent dimensions. Based on their composition, the dimensions were labeled as: FM symptoms and their characteristics (Dimension 1: “symptomatology”), familial and personal comorbidities (Dimension 2: “comorbidities”) and FM core clinical scales (Dimension 3: “scales”) (Figure 1, Table 1). The composition of the resulting dimensions was homogeneous: only four variables (trembling, personal history of chronic pain and SF-36 mental and physical subscales) clustered in apparently unrelated dimensions. Since their weights within the respective dimension were among the lowest, their effect on the subsequent FM classification was reduced.

The same analysis was performed on these variables in a second cohort of 887 patients, obtaining the same dimensions (Figure S2). Given this confirmation, a global analysis was performed using the whole cohort to obtain a global weight for each variable in order to perform patient classification.

**Figure 2:**
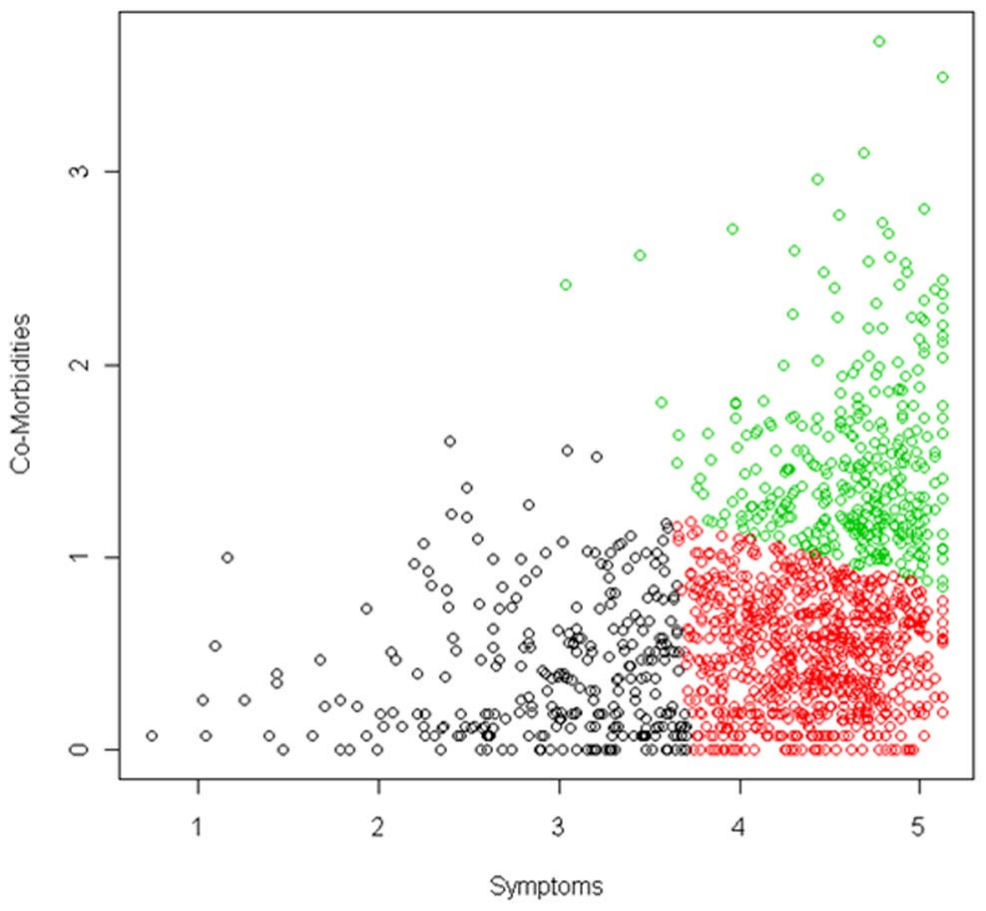
Subgrouping of fibromyalgia (FM) samples based on their “familial and personal comorbidities” (Y axis) and “FM symptoms and their characteristics” (X-axis) scores. Circles represent FM patients and are colored by cluster. Black circles for Cluster 1 (Low levels of symptoms and their characteristics and low levels of comorbidities), green circles for Cluster 2 (High levels of symptoms and high levels of comorbidities), and red circles for Cluster 3 (High levels of symptoms and low levels of comorbidities).

In order to maximize the number of samples classified into subgroups, it was decided to use only the two dimensions considered more reliable (“symptomatology” and “comorbidities”). On one hand, these dimensions included more variables with higher weights making them more reliable. In addition, we considered the possibility that the clustering of all clinical scales in a dimension could be due to the nature of the variables and not necessarily to their behavior in patients’ classification. This also allowed the subsequent use of core clinical scales for the assessment of the resulting subgroups and their disease severity. Using the scales in this way allowed us to maximize the information from the scales. For each FM sample, a composite index (score) was calculated for these two selected dimensions. The resulting scores were used to classify 1,398 out of the 1,446 FM samples. Cases were classified into three subgroups: low symptomatology and low levels of familial and personal comorbidities (Cluster 1; 283 cases (20.2%)), high symptomatology and high comorbidities (Cluster 2; 357 cases (25.6%)), and high symptomatology but low comorbidities (Cluster 3; 758 cases (54.2%)) (Figure 2).

The resulting FM subgroups presented no differences in terms of gender, age, marital status, and employment. However, patients having higher levels of education (high school degree and above) were more represented in cluster 1 than in the other clusters (p = 0.015 chi-square test).

This symptom-based classification correlated with the data from the scales measuring pain, fatigue, psychiatric symptoms, and their impact in life, as individuals belonging to the low symptomatology and low comorbidities group had lower medians for the scales (Table 2).

When the relationship between FM subgroups and core measures of severity was analyzed, Cluster 1 was markedly different from Cluster 2 and Cluster 3 in all scales, identifying the less affected group. Cluster 2 was more affected than Cluster 3, but differences were not as significant as with Cluster 1 in comparison with the other two groups (Table 2).

The FM subgroup with high pain and comorbidities was also the one with the highest level of fatigue (fatigue VAS higher than pain VAS) and, in fact, 20% of the patients in this cluster fulfilled also chronic fatigue syndrome (CFS) criteria [28] (FM+CFS), whereas in the other two clusters only 8 (Cluster 1) and 11% (Cluster 3) of the patients were fulfilling CFS criteria. Although pain VAS was the highest in this cluster, the number of tender points was not significantly different between both high pain clusters.

## Discussion

In this study we present a clustering of clinical data and subsequent FM subgrouping in a large cohort of FM patients. These findings were replicated in a second larger cohort, thus conferring a stronger robustness to our results. To our knowledge, this is the first study to perform a two-step clustering process to define variables’ dimensions and subsequently identify FM subgroups. The inclusion of personal and family history of comorbidities and the collection of data through direct physician examination constitute also novel contributions to FM cluster analysis.

Based on our data, the variables were grouped into three dimensions: FM symptoms and their characteristics (Dimension 1), familial and personal comorbidities (Dimension 2) and scales (Dimension 3). This clustering of variables into different dimensions within FM syndrome is in agreement with previous works [15]. The resulting variables dimensions are not completely unexpected, as some of the observed clustering can be attributed to the variables referring to the same symptom or organ (e.g. muscular symptoms in dimension 1).

A novelty of our findings is that pain symptoms were grouped with the cognitive symptoms into the first dimension. Since symptoms within a cluster may share a common identifiable etiology [8], this clustering could be highlighting the central nervous system implication in the physiopathology of FM [29]. Nevertheless, previous studies did not show the clustering of FM core symptoms and cognitive symptoms. In some cases, cognitive symptoms were not considered [2], [12], [13], or physical and psychological symptoms were considered separately [7], but in the study by Rutledge *et al*. (2009) [15], where both were considered, pain and cognitive symptoms did not cluster together. A possible explanation for these contradictory results is that in this study the evaluation was focused on the patients’ management of the symptoms and which ones the patients wanted to be improved, and not only the presence of the symptom. Another possible explanation could be that their data were collected in a different way, through online questionnaires instead of through a physician’s interview. The clustering of family history and personal comorbidities into a second dimension is also consistent with the fact that a family history of FM is linked with a more severe disease with more comorbidities [30]. Although this is an interesting finding of our study, it also constitutes a limitation, in that we cannot discriminate the relative weight of personal and family history of comorbidities, since they cluster together. However, personal history of chronic pain before extensive pain clustered in the first dimension, although presenting a low weight in the dimension. The fact that the history of chronic pain did not cluster with other comorbidities could be indicating that it may be considered as a supporting FM core symptom. In fact, it is difficult to identify the real onset of fibromyalgia, and whether the previous regional chronic pain belongs to the disease itself.

Finally, the cluster of scale-type variables in the third dimension could be due to the nature of the variables themselves rather than their clinical value. In fact, in order to cluster the scales with the dichotomous variables, they were transformed into binomial variables, which could have limited their clinical value. However the dichotomization of these variables improved the results by providing different meaningful clusters based on the symptoms and comorbidities variables, which were all them dichotomous. In any case, this last dimension was the one with the fewer variables and the lowest weights, making the dimension less reliable than the other two. Since the main purpose of clinical scales is to measure severity (constituting also a screening method), they were finally used to evaluate differences among the resulting FM subgroups.

Once the clustering of the samples was performed based on their scores in the first and second dimension only, the samples formed three groups: low symptomatology and low familiar and personal comorbidities (Cluster 1), high symptomatology and high familiar and personal comorbidities (Cluster 2) and high symptomatology but low familial and personal comorbidities (Cluster 3). Nevertheless, as shown in Figure 2, these three groups were not clearly separated, and the cut-offs used were empirically derived from our data (by k-means clustering method), and would be dependent of the specific cohort analysed.

We did not observe differences in age or gender among the subsets. It has to be taken into account that due to the reduced number of males included in the study (49 individuals), we were underpowered to exclude a possible difference in the distribution of males and females among subgroups. In agreement with previous works [7], [9], we found no age differences between the subgroups, neither in age at the time of recruitment nor in the age of onset. We did observe that patients included in Cluster 1 showed markedly lower values for all scales (Table 2). This could be pointing to a milder form of FM, or to a less evolved disease. However, we evaluated time of evolution of the disease and did not observe statistical differences between the three clusters, although patients belonging to Cluster 2 seemed to have a longer disease evolution (Table 2). This would indicate that the differences among the clusters are not related to disease staging, and could point to the identification of different clinical subsets. A prospective study would be useful to elucidate if the less affected Cluster 1 could have a better prognosis.

FM patients belonging to Cluster 2 (high symptomatology and high comorbidities) were also the ones with the highest levels of pain. This should not be unexpected, as most comorbidities are psychiatric, and individuals with pain have been shown to be more prone to depression because of pain’s adverse effects on mood and physical function [8]. Furthermore, previous cluster analyses showed that depression, fatigue and pain were all significantly correlated to each other (and to total health status) in persons with breast cancer [31], and that the number of depressive symptoms was a factor associated with the development of chronic widespread pain [32]. Comorbid medical conditions could also be responsible for a greater severity of FM symptoms in Cluster 2. However, differences between Clusters 2 and 3 in FM core measures observed in our study, although statistically significant, were limited. This could be indicative of a limited influence of personal or family history of chronic pain and psychopathology in disease severity, showing that the presence of high number of symptoms is the main marker of disease severity.

The FM subgroups that arise from our study are similar to the ones described in previous works [7], [10], [12]. In the analysis presented by Giesecke *et al*. (2003) [12], they also identified three subsets of patients, mainly based in pain and psychopathology: 1) moderate mood ratings, moderate levels of catastrophizing and perceived control over pain and low levels of tenderness; 2) elevated mood ratings, high levels of catastrophizing and low level of perceived control over pain and high levels of tenderness; and 3) normal mood ratings, low levels of catastrophizing and high level of perceived control over pain and extreme tenderness. Apparently, their groups show similarities with our findings, despite analyzing different variables. Nevertheless, our results and the results presented in Giesecke *et al.* (2003) [12] results are not directly comparable, as they used measurements of experimental pain and some variables not included in our study. Also, comparison of our results with those presented in the study by Rehm *et al.* (2010) [10] is difficult, as they use specific pain characteristics that were not evaluated in our work. Finally, in the study by Wilson *et al*. (2009) [7], they conducted a cluster analysis on the physical and psychological symptoms to identify subgroups. Subgroups I and IV of the classification in Wilson *et al.* (2009) [7] seem to correspond to the groups II and I of our classification, respectively. It would be interesting to test the degree of similarity between the resulting groups of each study. In their study, as in the present study, the group of patients with lower levels of symptomatology had higher education levels. Differences in the study design (use of web-based surveys, and exclusion of personal and family comorbidities) could explain the resulting four groups instead of three. However, both Wilson *et al*. (2009) and the present study highlight the importance of symptoms in FM patient classification. This is, indeed, in agreement with the new diagnostic and classification criteria of fibromyalgia [3]. One of the weaknesses of comparing classification methods of FM patients is that a different set of variables is considered in each of the studies, and each combination of questionnaires is likely to yield its own subgroups. Nevertheless, comparison of classifications of different studies cannot be properly performed without actually applying all the analysis to the same set of patients and directly comparing the composition of each of the resulting subgroups. In fact, it would be of interest to find a consensus regarding criteria for subgrouping of fibromyalgia patients. Performing the various types of classification in the same set of patients could help in identifying the most relevant criteria. These subgroups could then be reproduced in different patient populations and used for response analysis in future trials.

In summary, we have built latent dimensions based on routinely collected clinical data, which have allowed us to distinguish three subsets of FM patients that show differences in severity of disease. Our results indicate that the evaluation of personal and family history of FM comorbidities can add important information to FM classification based on somatic symptoms. It is possible that the subgroups identified may show different prognosis or response to intervention, but this has not been analyzed, and further studies in this area would be of interest. Furthermore, the definition of clinically homogeneous FM subgroups constitutes a key step for research purposes leading to a better understanding of the biological basis of FM.

## Supporting Information

Figure S1 **Frequency plots of the quantitative variables.**
(DOC)

Figure S2 **Silhouette plots for the analysis of the 3 different subsets.** A) silhouette plot for the first variable cluster analysis in 559 patients. B) silhouette plot for the variable cluster analysis in 887 patients. C) silhouette plot for the analysis of the whole set of patients. The variables clustering into each dimension are practically the same; only one variable, with the lowest importance in the composite of its assigned dimension (age of onset) shifted dimensions between B and C, and three variables (tender points, SF-36 physical and SF-36 mental), also each with the lowest importance in their assigned dimensions, shifted between A and C (data not shown).
(DOC)

Methods S1 **Linear functions for score calculation.**
(DOC)

List S1 **List of variables included in the cluster analysis of the clinical features of fibromyalgia listed in alphabetical order.** For continuous variables, median and 25 and 75 percentiles are included. Remaining variables were dichotomous (yes/no).
(DOC)
