# Supplementary figures and images for: Cluster Analysis of Clinical Data Identifies Fibromyalgia Subgroups
Source: PLoS One. 2013 Sep 30;8(9):e74873. doi: 10.1371/journal.pone.0074873 (PMC3787018; doi:10.1371/journal.pone.0074873)

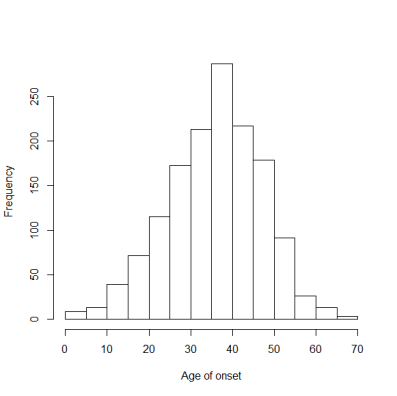

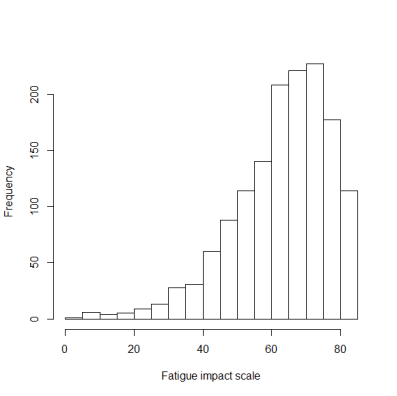

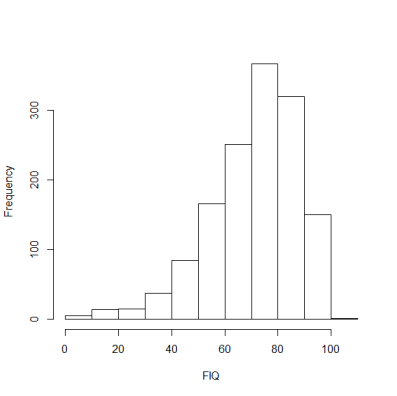

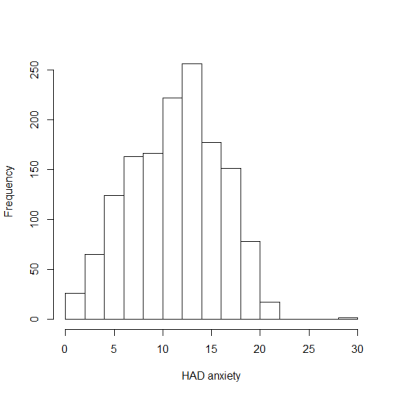

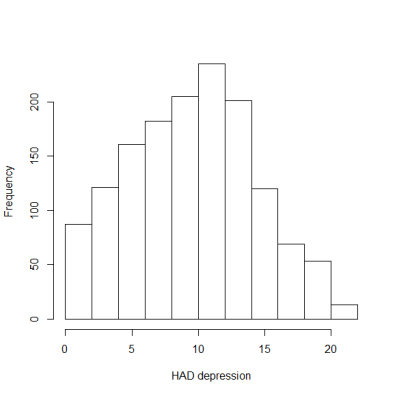

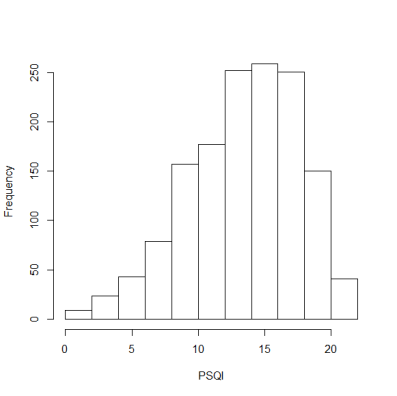

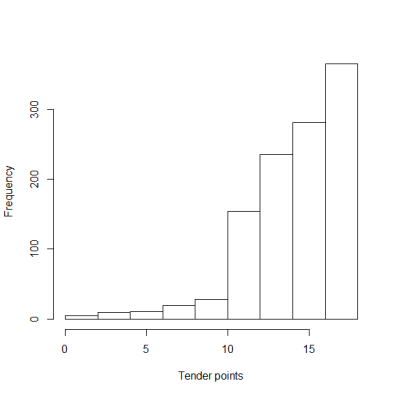

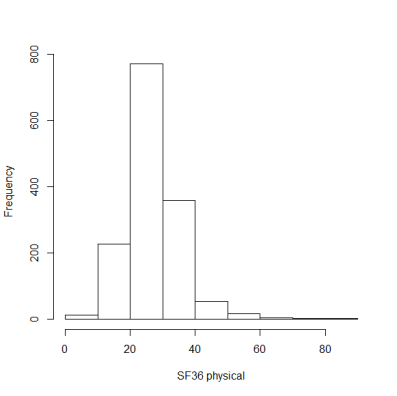

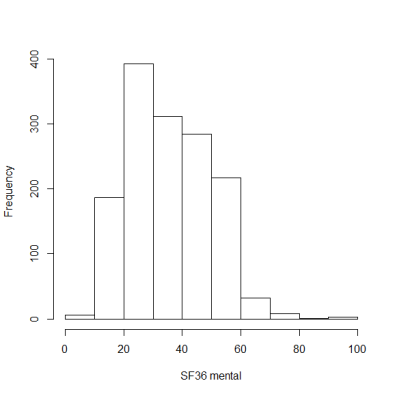

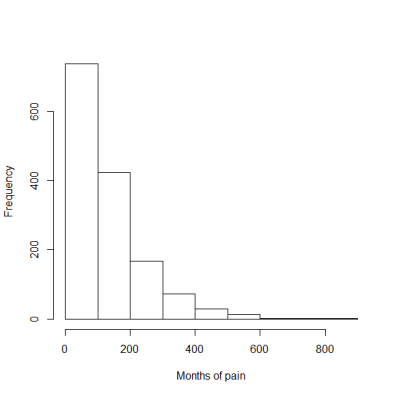

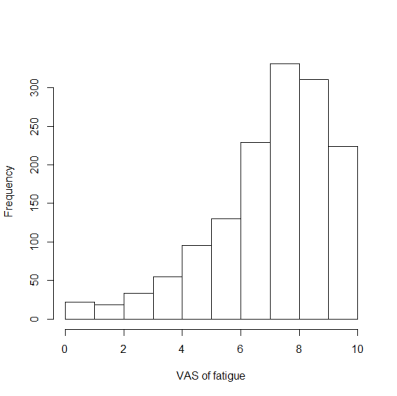

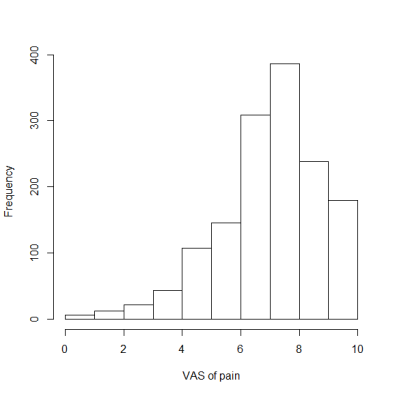


**Figure S1:** Frequency plots of the quantitative variables.

Supplement: Figure S1 — Frequency plots of the quantitative variables. (DOC) [file pone.0074873.s001.doc]
