# Supplementary material for: Cluster Analysis of Clinical Data Identifies Fibromyalgia Subgroups
Source: PLoS One. 2013 Sep 30;8(9):e74873. doi: 10.1371/journal.pone.0074873 (PMC3787018; doi:10.1371/journal.pone.0074873)
Supplement: Figure S2 — Silhouette plots for the analysis of the 3 different subsets. A) silhouette plot for the first variable cluster analysis in 559 patients. B) silhouette plot for the variable cluster analysis in 887 patients. C) silhouette plot for the analysis of the whole set of patients. The variables clustering into each dimension are practically the same; only one variable, with the lowest importance in the composite of its assigned dimension (age of onset) shifted dimensions between B and C, and three variables (tender points, SF-36 physical and SF-36 mental), also each with the lowest importance in their assigned dimensions, shifted between A and C (data not shown). (DOC) [file pone.0074873.s002.doc]

**A
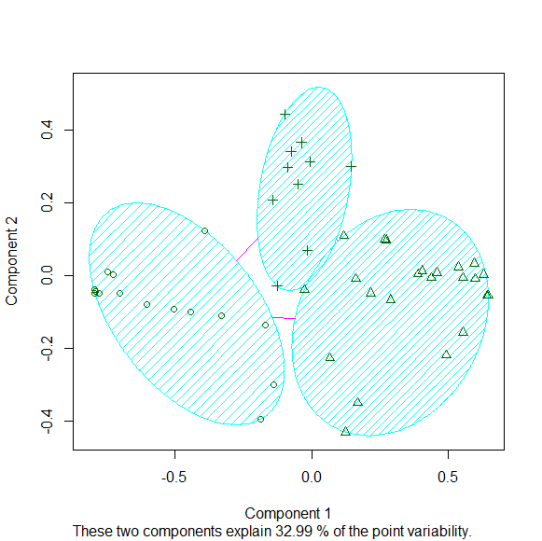
B
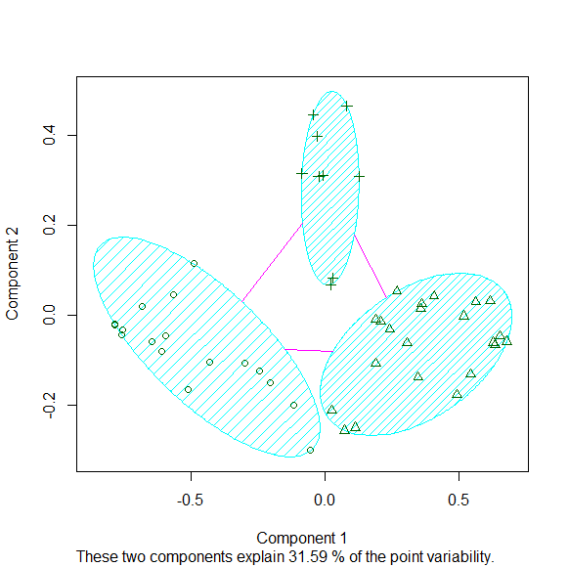
**

**C
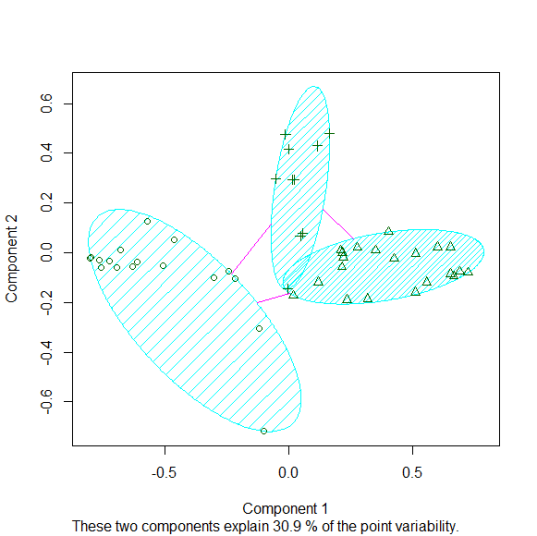
**

**Figure S2:** silhouette plots for the analysis of the 3 different subsets. A) silhouette plot for the first variable cluster analysis in 559 patients. B) silhouette plot for the variable cluster analysis in 887 patients. C) silhouette plot for the analysis of the whole set of patients. The variables clustering into each dimension are practically the same; only one variable, with the lowest importance in the composite of its assigned dimension (age of onset) shifted dimensions between B and C, and three variables (tender points, SF-36 physical and SF-36 mental), also each with the lowest importance in their assigned dimensions, shifted between A and C (data not shown).
