# Supplementary material for: Cluster Analysis of Clinical Data Identifies Fibromyalgia Subgroups
Source: PLoS One. 2013 Sep 30;8(9):e74873. doi: 10.1371/journal.pone.0074873 (PMC3787018; doi:10.1371/journal.pone.0074873)
Supplement: Methods S1 — Linear functions for score calculation. (DOC) [file pone.0074873.s003.doc]

**Methods S1**

Linear functions for score calculation:

Dimension 1

**Symptoms and their characteristics=** 0.417062 x Widespread pain + 0.403494 x Muscle weakness + 0.401630 x Post exercise fatigue + 0.386802 x Morning stiffness + 0.370318 x Muscular contractures + 0.363702 x Concentration problems +0.343472 x Memory complaints + 0.327471 x Onset + 0.295485 x Sleep Disturbances + 0.232666 x Forgetfulness + 0.232439 x Migratory joint pain + 0.196143 x Headache + 0.192320 x Pain subtle movements impairment + 0.188124 x Intestinal dysfunction + 0.134756 x Visual accommodation impairment + 0.128889 x Trigger + 0.106305 x Dizziness + 0.10344 x Excessive Perspiration +0.097555 x Months of pain + 0.081029 x Personal history of chronic pain + 0.079645 x Palpitations + 0.039180 x Age of onset

Dimension 2

**Personal and family comorbidities=** 0.574638 x Posttraumatic stress disorder +0.573018 x Personality disorders + 0.555378 x Family history of autoimmune disorders + 0.554531 x Family history of chronic fatigue syndrome + 0.491861 x Panic attacks + 0.466244 x Family history of fibromyalgia + 0.456721 x Blackouts + 0.432674 x Facial oedema + 0.395716 x Connective disorder + 0.363677x Adjustment disorder + 0.321596 x Previous Personal history psychopathology+ 0.295372 x Major depression + 0.188152 x Family history of chronic pain + 0.155414 x Impaired urination + 0.116031 x Spine osteoarthritis + 0.071334 x Life quality SF-36 physical subscale + 0.004091 x Life quality SF36 mental subscale
