# Supplementary material for: Cluster Analysis of Clinical Data Identifies Fibromyalgia Subgroups
Source: PLoS One. 2013 Sep 30;8(9):e74873. doi: 10.1371/journal.pone.0074873 (PMC3787018; doi:10.1371/journal.pone.0074873)
Supplement: List S1 — List of variables included in the cluster analysis of the clinical features of fibromyalgia listed in alphabetical order. For continuous variables, median and 25 and 75 percentiles are included. Remaining variables were dichotomous (yes/no). (DOC) [file pone.0074873.s004.doc]

**List S1.** List of variables included in the cluster analysis of the clinical features of fibromyalgia listed in alphabetical order. For continuous variables, median and 25 and 75 percentiles are included. Remaining variables were dichotomous (yes/no).

|  |
| --- |
| Adjustment disorder |
| Age of onset (38;p_25_: 30; p_75_:45) |
| Blackouts |
| Concentration problems |
| Connective disorder |
| Dizziness |
| Excessive Perspiration |
| Facial oedema |
| Family history chronic pain |
| Family history of autoimmune disorders |
| Family history of chronic fatigue syndrome |
| Family history of fibromyalgia |
| Fatigue Impact Scale (FIS) (66; p_25_:56.50; p_75_:75.00) |
| Fatigue level (VAS 1-10 cm) (8; p_25_:6.4; p_75_: 9) |
| Fibromyalgia Impact Questionnaire (FIQ) (74.66; p_25_:63.05; p_75_:84.25) |
| Forgetfulness |
| HAD anxiety subscale (12; p_25_:8; p_75_:15) |
| HAD depression subscale (10; p_25_:7; p_75_:14) |
| Headache |
| Impaired urination |
| Intestinal dysfunction |
| Life quality SF36 mental subscale (35; p_25_:25; p_75_:48) |
| Life quality SF-36 physical subscale (27; p_25_:22; p_75_:32) |
| Major depression |
| Memory complaints |
| Migratory joint pain |
| Months of pain (96; p_25_:48; p_75_:156) |
| Morning stiffness |
| Muscle weakness |
| Muscular contractures |
| Number of tender points (13; p_25_:13; p_75_:18) |
| Onset type |
| Pain level (VAS 1-10 cm) (7.5; p_25_:6.5; p_75_:8.5) |
| Pain subtle movements impairment |
| Palpitations |
| Panic attacks |
| Personal history of chronic pain |
| Personality disorders |
| Pittsburgh Sleep Quality Index (PSQI) (14; p_25_:11; p_75_:17) |
| Post exercise fatigue |
| Posttraumatic stress disorder |
| Previous Personal history psychopathology |
| Sleep Disturbances |
| Spine osteoarthritis |
| Trembling |
| Trigger Presence |
| Visual accommodation impairment |
| Widespread pain |
